# Does spiked tibial cement spacer reduce spacer-related problems in two-stage revision total knee arthroplasty for infection?

**DOI:** 10.1007/s00264-022-05438-6

**Published:** 2022-05-18

**Authors:** Kwang-Hwan Jung, Chae-Chil Lee, Tae-Hoon Kim, Jung-Won Han, Ki-Bong Park

**Affiliations:** Department of Orthopedic Surgery, Ulsan University Hospital, University of Ulsan College of Medicine, 877 Bangeojinsunhwan-doro, Dong-gu, Ulsan, 44033 Republic of Korea

**Keywords:** Knee, Infection, Revision, Total knee arthroplasty, Cement spacer, Spiked

## Abstract

**Purpose:**

Articulating cement spacers are frequently used in staged approaches for infected total knee arthroplasty (TKA). This study investigated whether a tibial cement spacer (TCS) with spikes could reduce spacer-related problems in two-stage revision TKA (R-TKA).

**Methods:**

A total of 27 patients (27 knees; 10 men and 17 women) who underwent two-stage R-TKA for infected TKA were retrospectively analyzed. Group A comprised 12 patients who used TCS with spikes added to the bottom surface, whereas group B consisted of 15 patients who used conventional TCS with a flat bottom. For each group, plain radiographs were obtained after cement spacer implantation and before R-TKA to measure mediolateral (ML) translation and TCS’s tilting angle. Patients’ demographic data, ML translation of the TCS, and changes in the TCS’s tilting angle between the groups were analyzed.

**Results:**

The mean ML translation was significantly lower in group A than that in group B (1.7 mm vs. 5.4 mm, *p* = 0.04). The mean change in the tilting angle was significantly lower in group A than that in group B (4.5° vs. 19.4°, *p* = 0.047).

**Conclusion:**

The spiked TCS in two-stage R-TKA provides superior stability compared to the TCS with a conventional design.

## Introduction

Articulating cement spacers (ACSs) are frequently used in staged approaches for infected total knee arthroplasty (TKA). As reported in the current literature, ACSs provide better mobility, reduced scarring, and better long-term range of motion (ROM) relative to the static design by providing structure and function similar to those of real arthroplasty implants [1, 2]. Furthermore, ACSs have a high infection control rate (96–98%), as well as reasonable function and satisfaction scores [3, 4]. Nonetheless, mechanically, the cement spacer can migrate, the knee joint can dislocate, and both the cement spacer and surrounding bone can fracture.

The features of cement spacers on follow-up radiographs include bone loss, spacer migration, spacer fracture, knee dislocation, and periprosthetic fracture [1]. Mediolateral (ML) translation and tilting of the spacer are considered minor problems, whereas spacer fracture, spacer dislocation, knee dislocation, and periprosthetic fracture are classified as major problems [5, 6]. A retrospective analysis of patients who underwent two-stage revision TKA (R-TKA) for infected TKA revealed that ML translation and spacer tilting were the most frequent spacer-related problems [5]. Although most spacer-related problems were deemed to be minor, 12% of patients exhibited major problems, including spacer fracture, spacer dislocation, or knee subluxation. Lau et al. [7] evaluated the effect of ACS subluxation on bone defects and the degree of constraint in R-TKA and reported that subluxation of the cement spacer was associated with the need for more constrained components at the time of reimplantation. It is logical to assume that these spacer-related problems can compromise the outcomes after two-stage R-TKA.

Previous studies introduced various methods for the prevention of spacer-related problems. Essentially, meticulous surgical techniques and regular monitoring of the cement spacer should be performed to avoid subluxation after cement spacer implantation [7]. In the case of a tibial cement spacer (TCS), the creation of an intramedullary stem during cement molding may aid in reducing cement bone interface motion; however, in some cases, the TCS has separated and migrated anteriorly from the anchoring cement and intramedullary post [8]. Recently, a handmade inverse cement spacer was developed to prevent cement spacer subluxation and fracture [6]. From our own clinical experience with ACS, we recognize that ML translation and tilting of the spacer mainly occur in the TCS. Dissimilar to a femoral cement spacer (FCS), which is in polygonal contact with the anterior femur, posterior femur, anterior chamfer, and posterior chamfer cutting surfaces, the TCS is in flat-on-flat contact with the tibial cutting surface. Therefore, we sought a way to increase the resistance to the force in order to move the TCS during knee joint ROM. We hypothesized that if we increased the resistance by making several spikes on the bottom surface of the TCS, a modified TCS with spikes could reduce spacer-related problems in two-stage R-TKA.

Therefore, the present study aimed to investigate whether a modified TCS with spikes could reduce spacer-related problems such as ML translation or tilting of the TCS in prosthesis-free interval while maintaining similar infection control compared to the TCS with a conventional design.

## Materials and methods

### Patient cohort

This retrospective study reviewed ACSs implanted between May 2019 and December 2021 in patients with either confirmed or suspected infection of knee replacement prostheses. We decided which type of spacer to implant intra-operatively based on the amount of bone loss after prosthesis removal and the status of extensor mechanism. We chose ACS for patients with minimal bone loss and an intact extensor mechanism. Patients who underwent R-TKA after only one cement spacer implantation were included in this study. Those who underwent R-TKA after repeated cement spacer changes were excluded from the analysis.

A total of 27 ACSs were implanted in 27 patients (17 women and 10 men). The mean patient age was 74.0 (range, 60–85) years. Causative microorganisms were isolated in 20 knees; the causative agent of infection was unknown in seven knees (25.9%). The most common infecting organism was methicillin-sensitive *Staphylococcus aureus* (25.9%), followed by *Escherichia coli* (18.5%), *Staphylococcus agalactiae* (11.1%), methicillin-resistant *Staphylococcus aureus* (11.1%), and *Staphylococcus epidermidis* (7.4%). The mean time between the first stage of R-TKA (removal of all implants and cement, thorough debridement, and ACS insertion) and the second stage of R-TKA (reimplantation of a revision prosthesis) was 8.4 (range, 6.3–12.5) weeks.

### Spacer design

**Fig. 1:**
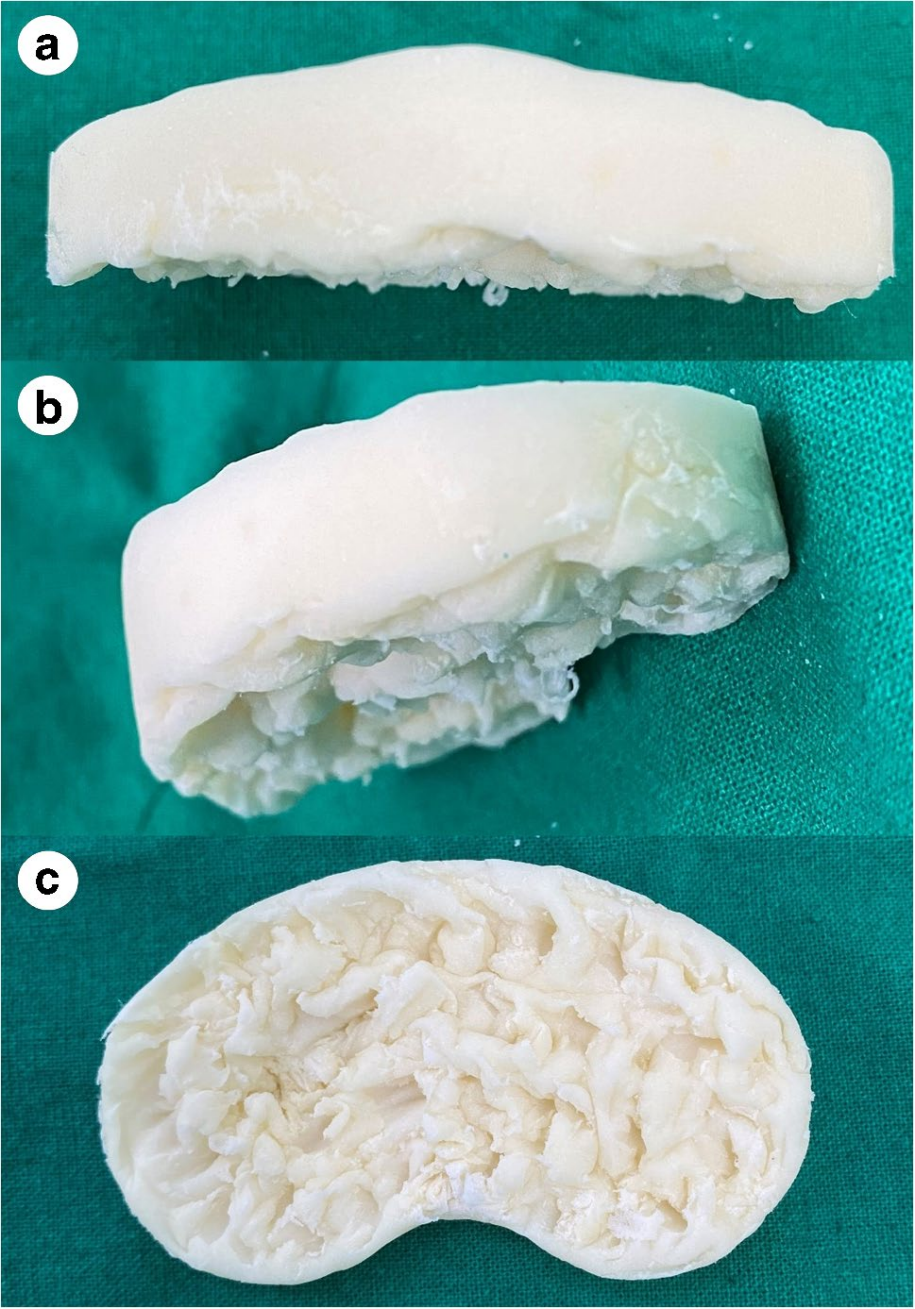
Intra-operative photograph of a tibial cement spacer with spikes. **a** Anteroposterior view. **b** Oblique view. **c** Bottom surface of tibial cement spacer

We chose a commercial bone cement (Simplex® HV with gentamicin; Stryker, Kalamazoo, MI, USA) that was based on poly(methyl methacrylate), had a powder-to-liquid ratio of 2:1, and had an antibiotic-to-bone cement ratio of 1:50 per package. If a specific culture organism was known pre-operatively, directed antibiotic choices were made; if the organism was unknown, the formula for antibiotics was based on 1 g of meropenem and 1 g of vancomycin per package. Additional antibiotics were mixed with bone cement containing 0.8 g of gentamycin sulfate. All cement spacers were molded intra-operatively using knee cement spacer molds, which allowed for FCS and TCS sizes ranging from 66 to 76 mm and 62 to 68 mm, respectively. Adequate thickness of the TCS was determined under traction of the leg at full extension and flexion of the knee joint at 90° to achieve gap balancing. Immediately prior to cement hardening, several spikes were made using a surgical probe on the bottom surface of the TCS (Fig. 1). In the control group with a flat bottom, the cement was naturally hardened and used without performing the aforementioned technique.

### Surgical procedures

A midline skin incision was made using the previous operative scar. A bacterial culture test was performed on the joint fluid while incising the joint using a medial parapatellar approach. Subsequently, the patellar tendon was carefully dissected so that patellar eversion could be achieved, even with a flexed knee. Synovial tissues were thoroughly debrided. First, the polyethylene insert was separated from the tibial component by using the manufacturer’s removal tool or a thin osteotome. Then, the firmness of the bone fixation of the femoral and tibial components was evaluated. If it was not firm, a thin osteotome was used; if it was firm, a saw was utilized to separate the bone cement interface. When the micro-movement of the components was confirmed, the components were re-moved after creating a see-saw phenomenon by hitting the component with a mallet. After removing all of the cement in the intramedullary canal, meticulous curettage was performed using a long curette until inflammatory tissues did not emerge. Next, both femoral and tibial intramedullary shafts were rinsed with 10 L of sterile normal saline. An assistant created a cement bead, which was to be placed into the intramedullary canal, while making the cement spacer using the above-described method. A cement about the size of a bean was made and was passed through a wire cut beforehand to create a cement bead. Before the cement in the mold was completely hardened, the mold was carefully separated, and the cement was shaped with some modifications so that the cement outlined the exposed bone surface. Afterward, the cement was left to harden completely. No cement was used to attach a cement spacer to the bone surface. A drain was placed, and the joint capsule and wound were closed layer by layer. Immediately after the operation, all patients were prescribed a knee immobilizer. Tolerable ROM exercises and non-weight-bearing ambulation were allowed in all patients until the second stage of R-TKA.

### Post-operative protocol

The patients had a two to eight week antibiotic-free interval following the completion of intravenous antibiotic regimen to allow for residual infection to re-emerge. This theoretically ensured that the samples collected at reimplantation for microbial culture were not falsely negative because of the previous antibiotic administration. Intra-operatively, in order to identify and quantify polymorphonuclear cells per high-power field, frozen sections were used as a decision-making tool for reimplantation, with reimplantation held if the results were positive. All patients underwent reimplantation with the Triathlon® revision system (Stryker, Kalamazoo, MI, USA). At the latest follow-up, none of the patients showed evidence of infection or required chronic antibiotic therapy.

### Laboratory evaluation

The white blood cell (WBC) count, erythrocyte sedimentation rate (ESR), and C-reactive protein (CRP) level were checked two weeks prior to R-TKA and were used to determine whether joint infection had been controlled between the two stages.

### Radiographic evaluation

Three fellowship-trained adult reconstruction surgeons conducted radiographic analysis for all anteroposterior and lateral radiographs acquired after cement spacer implantation. Radiographic evaluations were performed using the INFINITT picture archiving and communications system. The patients underwent radiography immediately after cement spacer implantation and at two weeks prior to R-TKA to evaluate the interval changes in the cement spacer. We evaluated ML translation and tilting angle of the TCS, spacer dislocation, and spacer fracture on each radiograph.

**Fig. 2:**
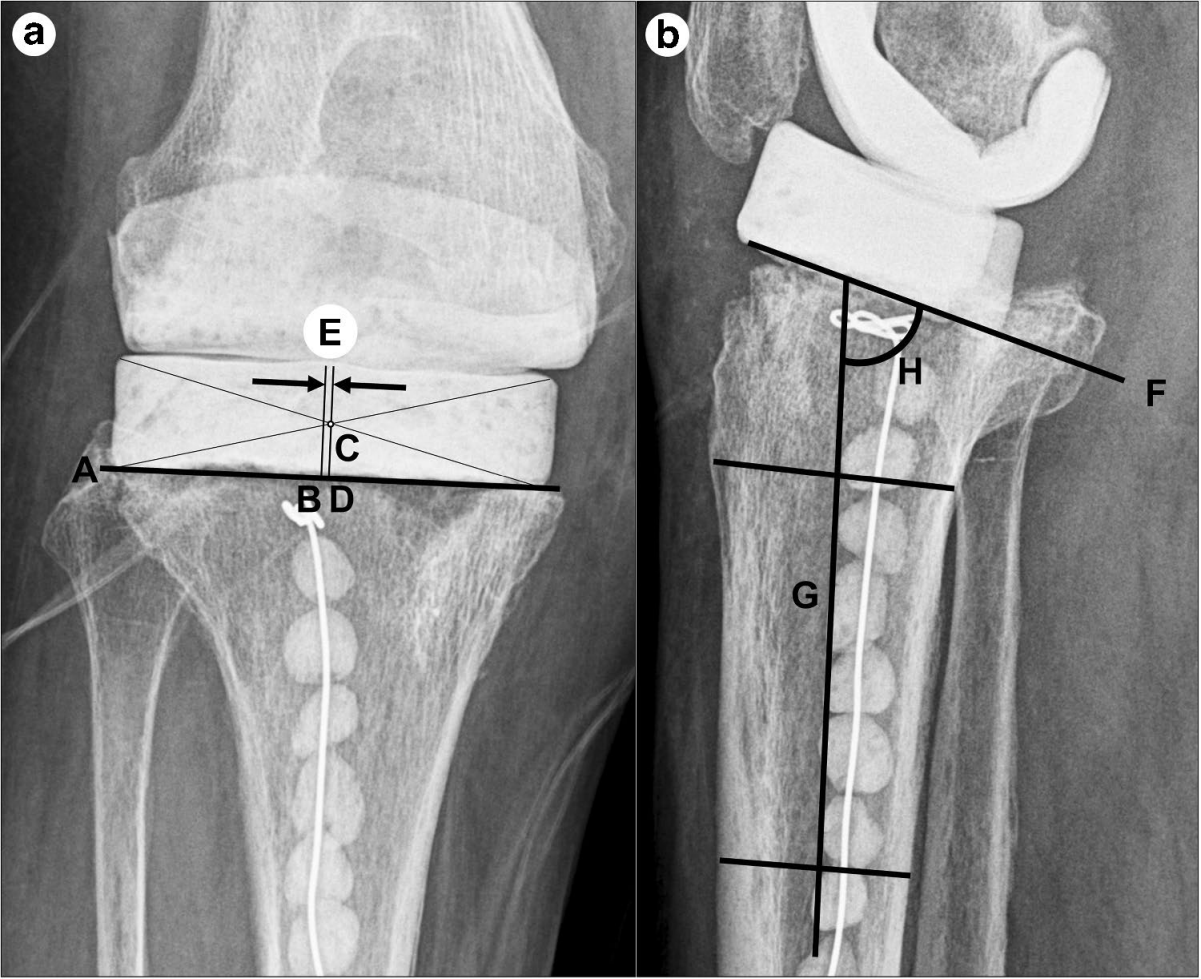
**a** Method of measurement of mediolateral translation of tibial cement spacer at the anteroposterior radiograph of the affected knee. **b** Method of measurement of tilting of tibial cement spacer at the lateral radiograph of the affected knee

As shown in Fig. 2a, the ML translation of the TCS was measured as follows. First, a line (A) parallel to the tibial cutting surface was drawn on the anteroposterior radiograph. Then, the center point (B) of line A was identified. Thereafter, the TCS was regarded as a rectangle, and the point (C) where two lines connecting the four vertices diagonally meet was taken as the centre point of the TCS. Subsequently, a vertical line was drawn from point C to line A, and the distance between the two points (D) was measured. As compared with point B, if point D was on the medial side, it was expressed as a positive number; if it was on the lateral side, it was expressed as a negative number. The difference (E) between the value of radiograph obtained immediately after the first stage of R-TKA and that of radiograph obtained just before the second stage of R-TKA was determined, and the value E was defined as the ML translation of the TCS.

As illustrated in Fig. 2b, the tilting angle of the TCS was measured as follows. First, a line (F) was drawn parallel to the bottom surface of the TCS on the lateral radiograph. Subsequently, the diaphyseal axis of the tibia was established as a line (G) connecting the two points equidistant from the anterior and posterior borders of the tibia: one was immediately inferior to the tibial tubercle, whereas the other was 10 cm distal to it. The angle (H) between these two lines was defined as the tilting angle of the TCS.

### Statistical analysis

All measurements are expressed as mean (range), and independent *t* tests were performed using SPSS for Windows version 11.5 (SPSS Inc., Chicago, IL, USA). Statistical significance was set at *p* < 0.05.

## Results

### Demographic findings

The characteristics of the two study populations are presented in Table 1. The female-to-male sex ratio was 8:4 in group A and 9:6 in group B (*p* = 0.50). The mean patient age was 74.5 (range, 63–85) years in group A and 73.5 (range, 60–81) years in group B (*p* = 0.96). No significant difference in the thickness of the polyethylene inserted in the previous primary TKA was identified (*p* = 0.49).

**Table 1:** Clinical characteristics of 27 patients who underwent two-stage revision total knee arthroplasty

	Group A (*n* = 12)	Group B (*n* = 15)	*p* value
Sex (female:male) ratio	8:4	9:6	0.50
Age (years)	74.5 (63–85)	73.5 (60–81)	0.96
Thickness of previous polyethylene insert (mm)	11.9 (11–14)	11.9 (10–15)	0.49
Pathogen			
*MSSA*	3	4	
*Escherichia coli*	2	3	
*Staphylococcus agalactiae*	2	1	
*MRSA*	1	2	
*Staphylococcus epidermidis*	0	2	
No growth	4	3	

*MSSA*, methicillin-sensitive *Staphylococcus aureus*; *MRSA*, methicillin-resistant *Staphylococcus aureus*

### Serological results

There were no statistically significant differences in WBC count, ESR, and CRP level between the two groups (Table 2).

**Table 2:** Comparison of WBC count, and ESR and CRP levels between the two groups

	Group A (*n* = 12)	Group B (*n* = 15)	*p* value
WBC count (/µL)	5550 (4320–8150)	6405 (2790–11,440)	0.41
ESR (mm/h)	33.0 (5–58)	42.7 (9–101)	0.17
CRP level (mg/dL)	0.31 (0.03–1.0)	0.48 (0.06–1.0)	0.87

*WBC*, white blood cell; *ESR*, erythrocyte sedimentation rate; *CRP*, C-reactive protein

### Radiographic results

**Fig. 3:**
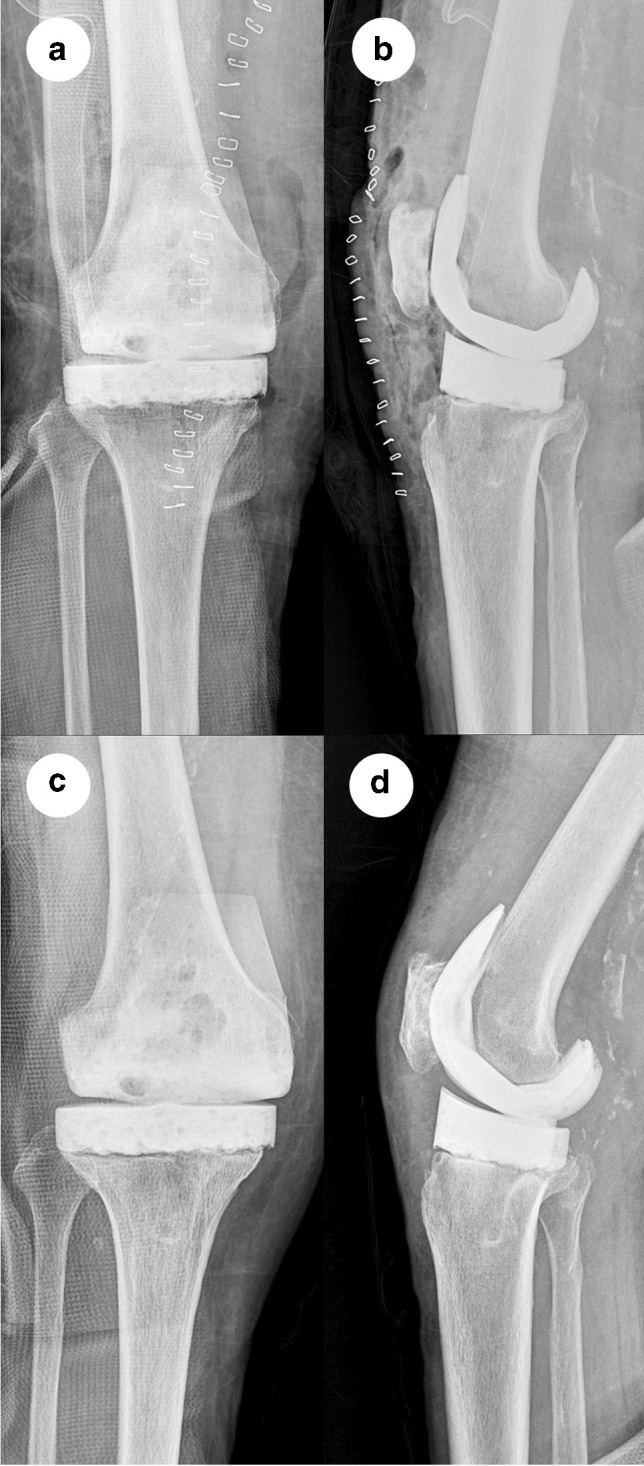
A 74-year-old woman with infected right knee prosthesis. Anteroposterior (**a**) and lateral (**b**) radiographs of the right knee after explanting the infected prosthesis and implanting the articulating cement spacer with spikes. Anteroposterior (**c**) and lateral (**d**) radiographs at 2 weeks prior to revision total knee arthroplasty. The interval changes in the tilting angle and mediolateral translation distance were 0.7° and 0 mm, respectively

**Fig. 4:**
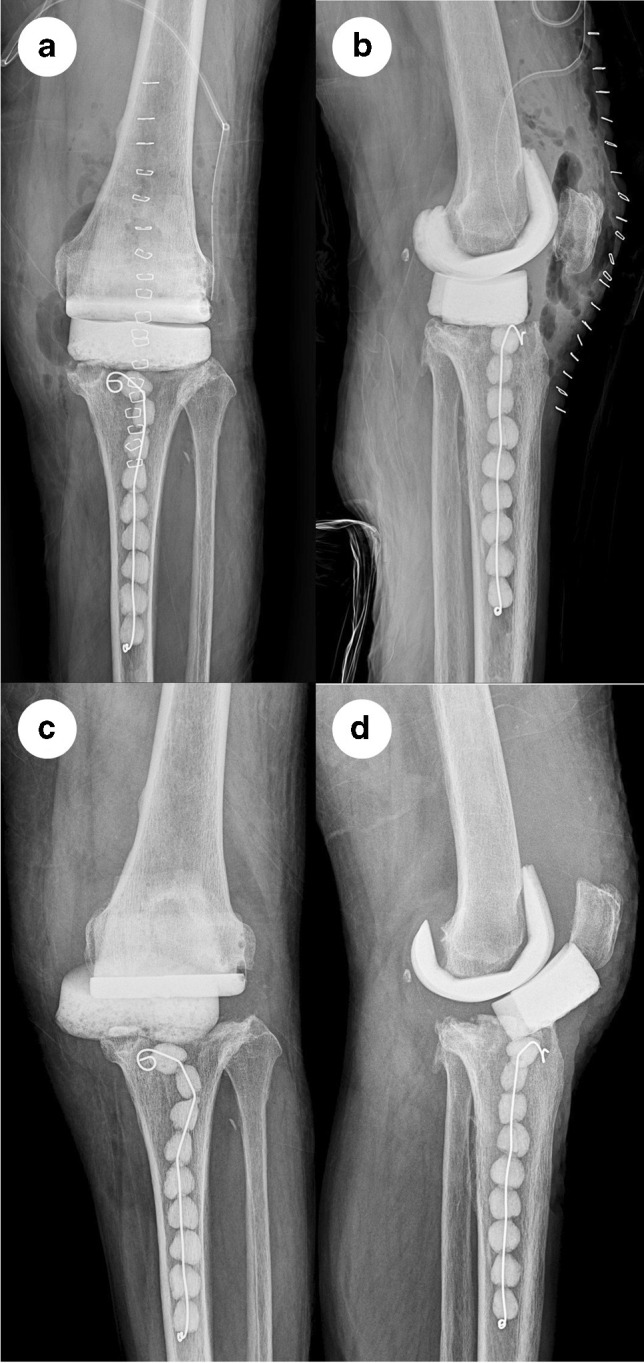
Anteroposterior (**a**) and lateral (**b**) radiographs of a 76-year-old woman after the first stage of revision total knee arthroplasty (TKA). Radiographs (**c**, **d**) prior to the second stage of revision TKA showed that the tibial cement spacer translated medially and tilted more

The radiographic results for the cement spacers are presented in Table 3 and Figs. 3 and 4.

**Table 3:** Comparison of radiographic findings between the two groups

	Group A (*n* = 12)	Group B (*n* = 15)	*p* value
TCS			
Tilting (°)	4.5 (0.1–32.1)	19.4 (0.6–35.8)	0.047
Mediolateral translation (mm)	1.7 (0.0–8.0)	5.4 (0.3–19.4)	0.01
TCS or FCS			
Spacer dislocation (*n*)	0	0	-
Spacer fracture (*n*)	0	1 (FCS)	0.07

*TCS*, tibial cement spacer; *FCS*, femoral cement spacer

The mean ML translation was 1.7 (range, 0.0–8.0) mm in group A and 5.4 (range, 0.3–19.4) mm in group B. The mean ML translation was significantly lower in group A than that in group B (*p* = 0.01).

The mean change in the tilting angle was 4.5° (range, 0.1–32.1) in group A and 19.4° (range, 0.6–35.8) in group B. The mean change in the tilting angle was significantly lower in group A than that in group B (*p* = 0.047).

In 27 patients, no dislocation of the spacer occurred during the interim period of two-stage R-TKA. In group A, there were no cases of spacer dislocation or spacer fracture. In group B, there were no cases of spacer dislocation; however, one patient had FCS fracture. Nevertheless, the rate of spacer fractures was not statistically significant (*p* = 0.07).

## Discussion

This study introduced a modified TCS that was intraoperatively supplemented with spikes on the bottom surface. The most important finding of this study was that modified TCS with spikes reduced spacer-related problems such as ML translation and change in the tilting of the TCS in prosthesis-free interval while maintaining similar infection control compared to the TCS with a conventional design.

Several parameters may play a role in spacer-related problems: spacer production (handmade, preformed, and surgical molds), spacer geometry, muscular sufficiency, prior surgeries, bone and soft-tissue quality (particularly the extensor mechanism), and patient non-compliance regarding partial weight-bearing [9]. In this study, because all cement spacers were manufactured using the same molds, there was no difference in spacer production. We believe that the technique of adding spikes to the bottom surface of the TCS resulted in a slight modification rather than changed the overall geometry of the spacer. As is known, the decision on which type of spacer to implant should be made based on the amount of bone loss and the status of extensor mechanism intraoperatively. If the bone loss is great or the quality of extensor mechanism is poor, it is recommended to select a static spacer in order to prevent post-operative dislocation [9]. In all patients included in this study, the extensor mechanism was confirmed intra-operatively and ACS was selected. Therefore, we estimated that there would be no difference in the occurrence of spacer-related problems between the two groups based on the difference in the status of the extensor mechanism. As all patients maintained non-weight-bearing during the prosthesis-free interval, we speculated that non-compliance related to partial weight-bearing would have little effect in this study.

As is well known [10], ACS can be classified according to fabrication techniques and constraint characteristics into cruciate-retaining (CR) and posterior-stabilized (PS) types. Lin et al. [11] compared the CR and PS types of ACS and reported that the overall mechanical complication rate was lower with PS spacers (9.3%) than that with CR spacers (45.5%). They assumed that the CR type without a post-cam construct could not counteract the mechanical force of the ML and anteroposterior directions and that the PS type with only a tibial post could provide ML constraint only, but not the anteroposterior direction. However, they used additional cement to implant cement spacers into the distal femur and proximal tibia. There were no statistically significant differences between the CR and PS types of ACS in terms of spacer loosening, spacer fracture, periprosthetic fracture, and extensor apparatus problems, except for joint dislocation which is most likely to be affected by the CR or PS type. Therefore, we think that it is necessary to interpret the prevalence of spacer-related problems by considering the effect of additional cement on cement spacer implantations.

The recent literature on spacer-related problems with ACS is summarized in Table 4 [5, 6, 12–16]. Previous studies reported the prevalence of spacer-related problems by dividing the participants into the conventional ACS group [5, 12–14] and modified ACS group [6, 15, 16]. Upon scrutinizing these previous studies, the prevalence of spacer-related problems was extremely low in the modified ACS group. This study showed that there was one case of FCS fracture (6.7%) in the conventional design group; in contrast, no cement spacer fracture occurred in the spiked design group. We assumed that when the patients with ACS performed knee joint ROM, micro- or macro-motion mainly occurred in the TCS because the TCS had relatively low stability between the cement spacer and bone surface. Additionally, we assumed that the forces in this motion of the TCS were concentrated on the relatively firmly attached FCS and that FCS fractures would via this mechanism.

**Table 4:** Summary of the types and prevalence of spacer-related problems according to types of cement spacer in previous studies

Type of cement spacer	Type of spacer-related problems	Prevalence
ACS		
*Struelens *et al*.* [5]	Suboptimal	57%
*Castelli *et al*.* [12]		4%
*Johnson *et al*.* [13]	Dislocation, subluxation, fracture	12%
*Lanting *et al*.* [14]	Anterior or posterior subluxation	89.4%
	Medial or lateral subluxation	66.3%
Modified ACS		
*Kim *et al*.* [15]		0%
*Hammerich *et al*.* [6]		0%
*Akhtar *et al*.* [16]		0% (vs. 84.6% without pedestal)

*ACS*, articulating cement spacer; *TCS*, tibial cement spacer

Recent studies have proposed various techniques for solving spacer-related problems. Hammerich et al. [6] introduced an inverse cement spacer for two-stage R-TKA and reported that 110 R-TKA did not show any indication for dislocation, subluxation, or fractures of the cement spacer on radiographs obtained before re-implantation. Tsai et al. [17] introduced an ACS with a computer-aided design for the treatment of periprosthetic knee infection and reported one case of a medial tibial plateau fracture with TCS tilting and one case of patellar maltracking disorder, with an overall complication rate of 6.3% (2 out of 32 cases). Akhtar et al. [16] described a cement pedestal spacer technique used in two-stage R-TKA and reported that there were no spacers with subluxation or tilting. However, they showed that ACS with no cement pedestal displayed the greatest number of complications (subluxation, tilting, and cement spacer fracture). Gililland et al. [18] pointed out that various ACSs could not tolerate full weight-bearing or provide adequate stability for daily function. They introduced a surgical technique for gap-balanced ACS using cement augmentation and dowel stems; nonetheless, they did not present the short- or long-term follow-up results after using this technique. Lo Presti et al. [19] conducted a retrospective study on 12 patients who underwent two-stage R-TKA using a static cement spacer with two intramedullary Küntscher nails. Four out of six patients who underwent several debridement procedures experienced at least one spacer dislocation after previous surgeries; however, the static cement spacer with Küntscher nails was used 16 times in 12 patients, and there were no cases of recurrent dislocation. Because the static cement spacer was used, all patients could not perform ROM exercises, and only toe-touch weight-bearing ambulation using crutches or a walker was allowed. In the present study, we reduced ML translation and amount of change in the tilting angle of the TCS, as compared with the conventional cement spacer, by adopting a simple technique of adding spikes to the TCS. There were only one cement spacer fracture and no spacer dislocations among the 27 patients.

This study had some limitations. First, this study included a small number of patients in each group. Second, data on the biomechanical characterization of the TCS with spikes could not be provided. Third, among the parameters that may affect the occurrence of spacer-related problems, muscular insufficiency, previous surgical revisions, and amount of bone loss could not be evaluated. Fourth, conventional TCS was implanted during the earlier period of this study, whereas modified TCS with spikes was implanted during the later period. This might have resulted in differences in the learning curve of surgeons and might have led to a lower mean ML translation and mean amount of change in the tilting angle of the modified TCS. Lastly, the effects of grading the ML translation or the change in the tilting angle of the TCS on clinical outcomes (e.g., ROM, pain scale) during the prosthesis-free interval remain to be determined. Nonetheless, this study focused on the level of infection control and the prevalence of spacer-related problems.

Patients who underwent two-stage R-TKA with spiked TCS showed lower mean ML translation and the mean amount of change in the tilting angle of the TCS than those in whom conventional TCS with a flat bottom was used. The spiked TCS in two-stage R-TKA is easy to fabricate and provides superior stability compared to the TCS with a conventional design.

## Data Availability

Not applicable.
